# Delivery during Sleep

**Published:** 1846-06

**Authors:** 


7. *Delivery during Sleep.*—M. Schultze, of Spandow, was called, on the 25th of May, 1844, to a woman pregnant for the fourth time, at full term, and whom he found in so profound a sleep that he could not rouse her, either by shaking her, or by the vapors of ammonia, ether, &c., applied to the nostrils. On the third day of this unnatural sleep, the female was delivered, without awakening, of a male child, at full term, alive and well. On his visit of the morrow, M. Schultze found that his patient had been awake for a short time; she had recovered of herself, and as she had no recollection of what had happened, she appeared much surprised to find herself safely delivered.—*Ibid.*

